# High cytidine deaminase expression in the liver provides sanctuary for cancer cells from decitabine treatment effects

**DOI:** 10.18632/oncotarget.597

**Published:** 2012-09-27

**Authors:** Quteba Ebrahem, Reda Mahfouz, Kwok Peng Ng, Yogen Saunthararajah

**Affiliations:** ^1^ Taussig Cancer Institute, Cleveland Clinic, Cleveland, OH

**Keywords:** cytidine analogues, decitabine, cytidine deaminase, chemotherapy resistance, sanctuary

## Abstract

We document for the first time that sanctuary in an organ which expresses high levels of the enzyme cytidine deaminase (CDA) is a mechanism of cancer cell resistance to cytidine analogues. This mechanism could explain why historically, cytidine analogues have not been successful chemotherapeutics against hepatotropic cancers, despite efficacy in vitro. Importantly, this mechanism of resistance can be readily reversed, without increasing toxicity to sensitive organs, by combining cytidine analogue with an inhibitor of cytidine deaminase (tetrahydrouridine). Specifically, CDA rapidly metabolizes cytidine analogues into inactive uridine counterparts. Hence, to determine if sheltering/protection of cancer cells in organs which express high levels of CDA (e.g., liver) is a mechanism of resistance, we utilized a murine xenotransplant model of myeloid cancer that is sensitive to epigenetic therapeutic effects of the cytidine analogue decitabine in vitro and hepato-tropic in vivo. Treatment of tumor-bearing mice with decitabine (subcutaneous 0.2mg/kg 2X/week) doubled median survival and significantly decreased extra-hepatic tumor burden, but hepatic tumor burden remained substantial, to which the animals eventually succumbed. Combining a clinically-relevant inhibitor of CDA (tetrahydrouridine) with a lower dose of decitabine (subcutaneous 0.1mg/kg 2X/week) markedly decreased liver tumor burden without blood count or bone marrow evidence of myelotoxicity, and with further improvement in survival. In conclusion, sanctuary in a CDA-rich organ is a mechanism by which otherwise susceptible cancer cells can resist the effects of decitabine epigenetic therapy. This protection can be reversed without increasing myelotoxicity by combining tetrahydrouridine with a lower dose of decitabine.

## INTRODUCTION

Cytidine analogue chemotherapeutics are rapidly metabolized into inactive uridine counterparts by the enzyme cytidine deaminase (CDA)[1-4]. The impact of CDA on the pharmacology of cytidine analogues is illustrated by the difference between *in vitro* and *in vivo* half-life: the half-life of decitabine in buffer *in vitro* at 37^°^C is >10 hours[5], by contrast, the half-life *in vivo* is <10 minutes[6], a drastic reduction largely attributable to CDA[2, 7-8]. Hence, it is possible that high expression of CDA in some organs, such as the liver, provides protection for malignant cells from the effects of cytidine analogues. However, such protection or sanctuary has not been formally evaluated as an actual mechanism of resistance to cytidine analogues, and there are no routine measures in place to reverse it, even though it could explain the historically poor responses of hepatotropic cancers treated with cytidine analogues[9-10].

The cytidine analogue drugs 5-azacytidine and decitabine have a therapeutic molecular epigenetic effect, depletion of DNA methyl-transferase 1 (DNMT1) (5-azacytidine is converted to decitabine by ribonucleotide reductase prior to DNA incorporation), at non-cytotoxic concentrations well below 0.5 μM[11-17]. Hence, in contrast to the cytidine analogues cytarabine and gemcitabine, which are administered at high dosage (100-3000 mg/m^2^) derived from maximum tolerated levels and intended for anti-metabolite cytotoxic effects, 5-azacytidine and decitabine are administered at relatively low dosage (5-75 mg/m^2^). These low dosages may be even more susceptible to failure caused by CDA-mediated degradation and sanctuary, limiting the clinical role of these unique epigenetic therapy agents.

The uridine analogue tetrahydrouridine (THU), a competitive inhibitor of CDA, has been used as a CDA inhibitor in combination with cytidine analogues pre-clinically and clinically for some decades, without documentation of toxic side-effects from THU[2-4, 8, 11, 18-25]. Sanctuary in a CDA-rich organ as an actual mechanism of resistance has not been evaluated, thus, neither has the ability of THU to reverse such sanctuary. For safe and practical clinical application, THU should improve distribution of cytidine analogue into the sanctuary organ but without increasing toxicity in sensitive tissues, for example, the bone marrow. Hence, the objectives of the present study were to evaluate if the liver, a CDA-rich organ, can function as a sanctuary site for cancer cells which are otherwise known to be sensitive to decitabine treatment effects, and furthermore, to determine if the addition of THU to the treatment regimen can reverse such sanctuary, and do so without increasing myelotoxicity. The myeloid cancer cell line THP1 was used for these experiments, since we have demonstrated its sensitivity to non-cytotoxic, DNMT1-depleting concentrations of decitabine *in vitro*, and its hepatotropism *in vivo*[26].

## MATERIALS AND METHODS

### Treatment of a xenotransplant model of hepatotropic malignancy with decitabine and tetrahydrouridine

Experiments were approved by the Cleveland Clinic Institutional Animal Care and Use Committees (IACUC). p53-null THP1 cells were purchased from ATCC (Manassas, VA). This morphologically monocytoid myeloid leukemia cell line contains an MLL-AF9 fusion, is homozygously mutated at the *TP53* and *CDKN2A* loci, and demonstrates hepatic tropism *in vivo*[26]. The cells used for xeno-transplantation were transfected to express luciferase. THP1 cells were transplanted by tail-vein injection (0.4 x10^6^/mouse) into non-irradiated 6-8 week old NSG mice. Mice were anesthetized with isofluorane before transplantation. Beginning on day 5 after inoculation, animals were treated with vehicle (PBS), subcutaneous decitabine 0.2 mg/kg 2X/week (Monday, Thursday), or intraperitoneal THU 4mg/kg 30 minutes before a lower dose of subcutaneous decitabine 0.1 mg/kg 2X/week (Monday, Thursday). Alternatively, animals were treated with PBS, subcutaneous decitabine 0.2 mg/kg 2X/week or with THU in combination with the same dose of decitabine. Animals were checked daily and were euthanized by an IACUC approved method for signs of distress. To anatomically localize THP-1 cells in living mice, the substrate D-Luciferin (15 mg/ml D-luciferin in sterile PBS (Promega) was injected intra-peritoneal into one mouse per treatment group and mice were imaged after 10 minutes using an IVIS-200 CCD camera imaging system (Xenogen, Alameda,CA).

### Measurement of CDA enzyme activity

Using a modification of previously published methods[27], conversion of cytidine into uridine by homogenized liver tissue at 37^°^C was measured by high performance liquid chromatography (HPLC). Liver tissue (1mg) was gently homogenized in 1ml iced reaction buffer supplemented with protease inhibitors. Reaction buffer of 0.1M Tris/HCL pH 7.5 (265μl) was added to 25 μl of homogenized liver tissue followed by addition of cytidine to a final concentration of 4.1 mM and 5-flourouridine (0.381 mM) as an internal control (5-fluoruridine is not metabolized by CDA). This reaction mixture was incubated at 37^°^C for 60 minutes and the reaction terminated by addition of 50μl of 1N hypochloric acid. Blanks used in calculations (described below) consisted of the above but with cytidine substrate added at the end of the 60 minute incubation period. After termination of reactions, protein was precipitated by addition of trichloroacetic acid (TCA, 2%). Supernatant (20μl) was injected for HPLC analysis with ammonium acetate (15mM) as the mobile phase with a flow rate of 0.35 mL/min through Xbridge^™^ OST C18, 2.5μm, 4.6×50mm column on Dionex UltiMate^®^ 3000 μ-HPLC system supported with *Chromeleon*® 7.1 chromatography dtata system (Dionex Corporation, Sunnyvale, CA). Retention time and peak area of uridine at 260nm were compared to the internal control for each injection. The average net uridine peak area of test minus blank was calculated for each test sample. Uridine known concentrations from 0.0 to 95.8μM were used to plot a standard curve to calculate uridine amount based on the net uridine peak area. CDA enzyme unit activity was defined as the amount of enzyme that produces 1μM of uridine in 1 minute, reported as mU enzyme activity per minute. A purified CDA enzyme (genotype A79A, specific activity of 308.9U/mg, gift of Professor Silvia Vincenzetti, Universita di Camerino, Italy) was used as a calibrator and for quality control. Multiple runs with known concentrations of uridine were used to confirm accuracy and precision, and confirmed between run variability of < 5%.

## RESULTS

### Malignant cells find sanctuary from decitabine in the liver

**Figure 1:**
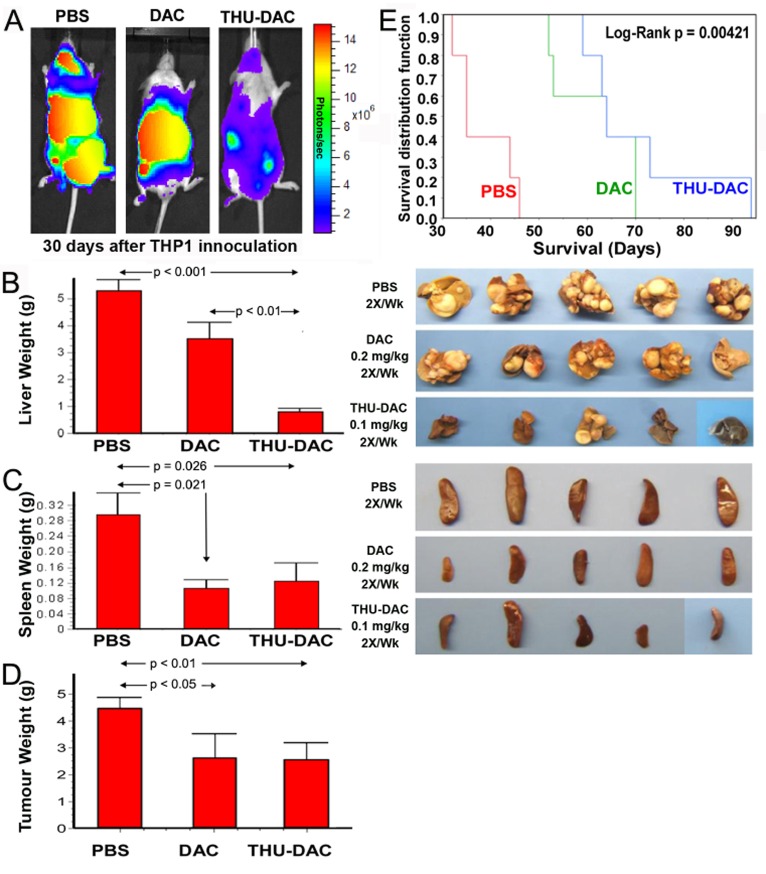
Both DAC alone and THU-DAC (DAC dose decreased to 0.1mg/kg) extended survival, however, combination with THU strikingly decreased hepatic tumor burden A) In vivo imaging on day 30 demonstrated wide-spread disease in PBS-treated mice, disease concentrated in the region of the liver in DAC-treated mice, and substantially less tumor burden in THU-DAC treated mice. THP1 myeloid leukemia cells were engineered to express luciferase. B) Liver appearance and weight in the different treatment groups. Livers were harvested at different time-points corresponding to the Kaplan-Meier curve. C) Splenic tumor burden was similarly decreased by DAC and THU-DAC compared to PBS. Spleens were harvested at different time-points corresponding to the Kaplan-Meier curve. Histogram shows spleen weights. p-value Wilcoxon test. D) Extra-splenic and extra-hepatic tumor burden was similarly decreased by DAC and THU-DAC compared to PBS. Most of this tumor burden was in DAC-treated mice intestines/peritoneum, another CDA-rich tissue. p-value Wilcoxon test. n=5/group. E) Kaplan-Meier plots for the different treatment groups. Mice were euthanized for signs of distress. n=5/group.

Mice were inoculated by tail-vein injection with 0.4×10^6^ THP1 myeloid cancer cells, which are sensitive to non-cytotoxic, DNMT1 depleting concentrations of decitabine *in vitro* and hepatotropic *in vivo*[26]. Beginning on day 5 after inoculation, animals were treated with vehicle (PBS), subcutaneous decitabine 0.2 mg/kg 2X/week, or a lower dose of subcutaneous decitabine 0.1 mg/kg combined with intraperitoneal THU 4mg/kg administered 30 minutes before the decitabine 2X/week (THU-decitabine)(n=5/group). Mice underwent euthanasia at different time-points determined by onset of signs of distress. Control PBS-treated mice demonstrated extensive disease in the regions of the liver, spleen and peritoneum observed by *in vivo* imaging of the luciferase-expressing THP1 cells on day 30, and by inspection/weighing of liver and spleen obtained after euthanasia for distress (Figure 1A-D). Treatment with decitabine significantly reduced the tumor burden in all sites (Figure 1A-D), and significantly extended median survival (61 days) compared to PBS (38 days, Log Rank p=0.0013) (Figure 1E). However, there remained strikingly substantial liver tumor (average 3.5 g in decitabine-treated mice compared to average > 5 g in PBS-treated mice) (Figure 1A-D).

### Combining THU with a lower dose of decitabine reversed sanctuary

Similar to treatment with decitabine alone, THU 4 mg/kg intraperitoneal combined with a lower dose of decitabine (0.1 mg/kg instead of 0.2 mg/kg) 2X/week extended median survival (70 days) compared to control PBS treatment (Log Rank p=0.00421, n=5/group) (Figure 1E). However, in comparison to mice treated with decitabine alone, combination THU-decitabine largely eliminated liver tumor burden (average 0.8 g in THU-decitabine treated mice compared to average 3.5 g with decitabine treatment, p<0.001) (Figure 1A, B). Extra-hepatic tumor burden was decreased by a similar extent by both decitabine and THU-decitabine (Figure 1C, D).

**Figure 2:**
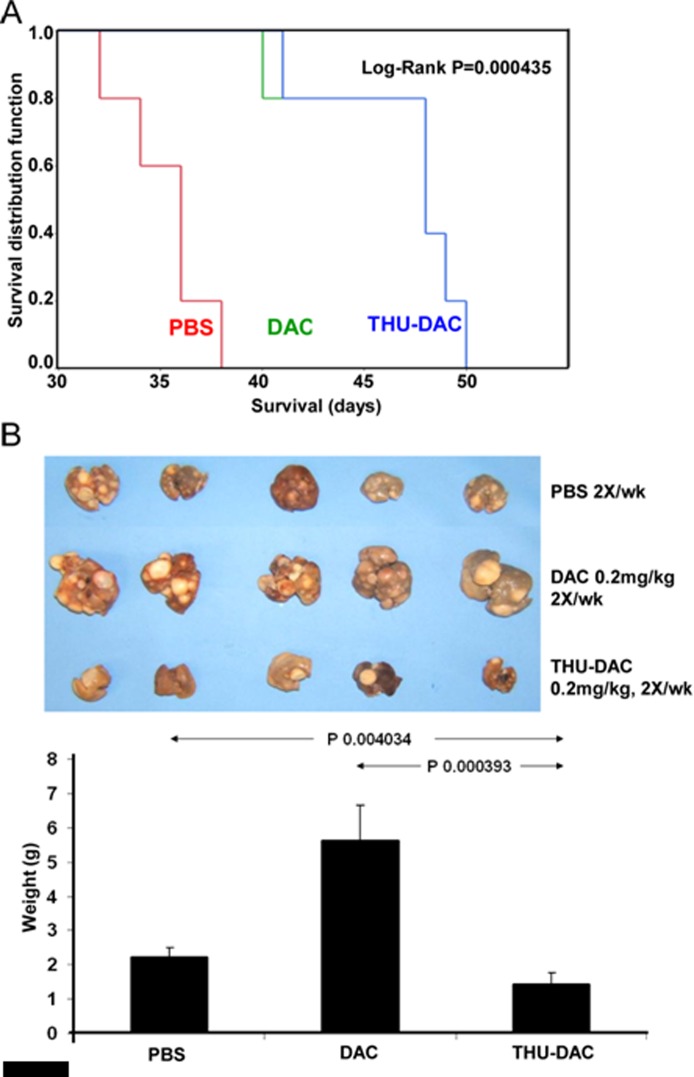
Mice treated with THU-DAC 0.2mg/kg 2X/week demonstrated similar survival to DAC 0.2mg/kg treated mice, despite a substantially decreased liver tumor burden, possibly because of myelotoxicity with this regimen in which the dosage of DAC was not reduced when combined with THU A) Kaplan-Meier plot. Mice were euthanized for signs of distress. The THP1 inoculum was 2 × 10^6^ cells. n=5/group. B) Liver appearance and weight in the different treatment groups. Livers were harvested at different time-points corresponding to the Kaplan-Meier curve (hence, the greater tumor burden in decitabine treated mice versus PBS treated mice). Histogram shows liver weights in grams.

These experiments were repeated using the same decitabine dose alone or in combination with THU (in the above experiments, the decitabine dose was lowered for combination with THU); treatment groups: (i) PBS 2X/week, (ii) subcutaneous decitabine 0.2 mg/kg 2X/week, (iii) subcutaneous decitabine 0.2 mg/kg 2X/week 30 minutes after intraperitoneal THU 4mg/kg (n=5/group). Again, the addition of THU substantially eliminated liver tumor burden, although this regimen, in which the dose of decitabine combined with THU was not decreased (Figure 2), did not increase survival compared to decitabine alone, possibly because of myelotoxicity (described below).

### Murine liver expresses substantially higher levels of CDA than murine spleen

**Figure 3:**
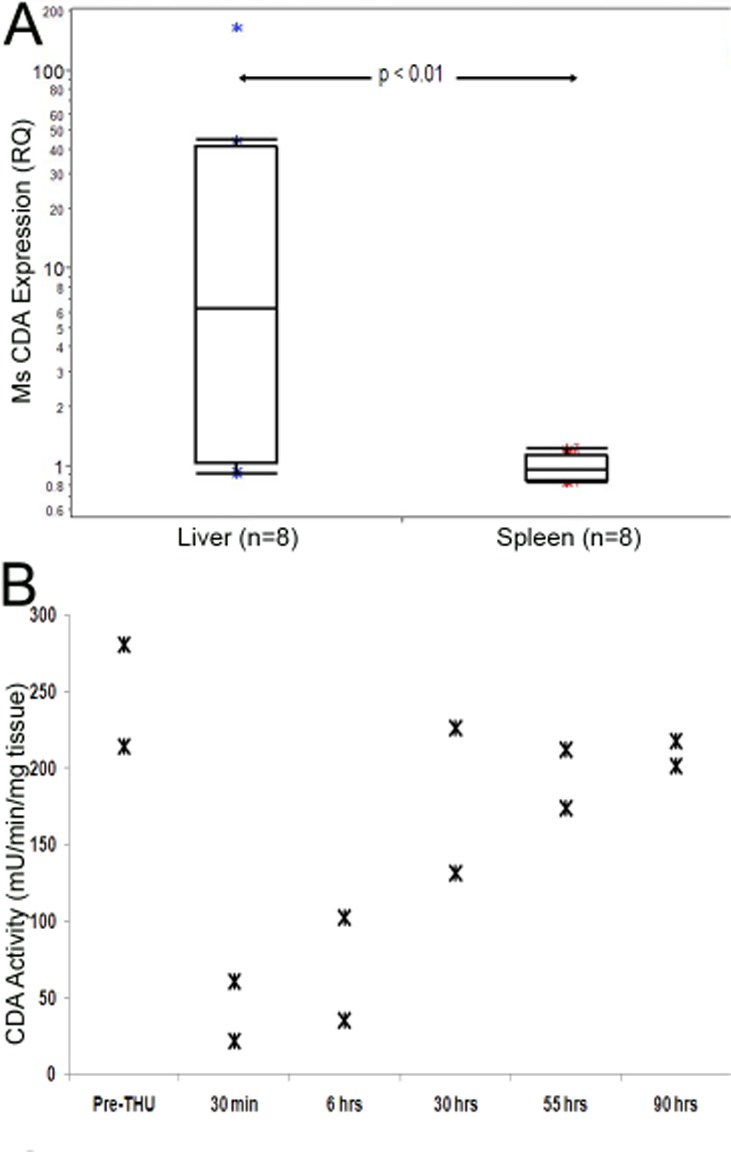
High CDA expression and activity in the liver, and inhibition of activity by tetrahydrouridine (THU) A) CDA expression is significantly higher in the liver versus spleen. CDA levels measured by QRT-PCR. P-value Wilcoxon test. B) THU temporarily inhibited liver CDA enzyme activity. A single dose of intraperitoneal THU 4 mg/kg alone was administered to 12 mice which were then sacrificed at staggered time-points after the THU administration for measurement of liver CDA enzyme activity by an HPLC assay.

The differences between liver and spleen in tumor burden and its reduction by decitabine alone suggested that CDA expression could be substantially higher in murine liver than in spleen. This was confirmed by QRT-PCR: CDA mRNA levels were significantly higher (>5-fold, p<0.01) in murine liver compared to spleen (Figure 3A).

### THU inhibits murine liver CDA enzyme activity

To confirm that THU inhibited liver CDA enzyme activity, THU 4 mg/kg alone was administered by the intraperitoneal route to 12 mice. These mice were sacrificed at staggered time-points after THU administration to measure liver CDA enzyme activity by an HPLC assay. A substantial reduction in liver CDA enzyme activity was observed 30 minutes and 6 hours after THU administration, with major recovery of this activity by 30 hours (Figure 3B).

### THU reversed cancer cell sanctuary in the liver without causing myelotoxicity

**Figure 4:**
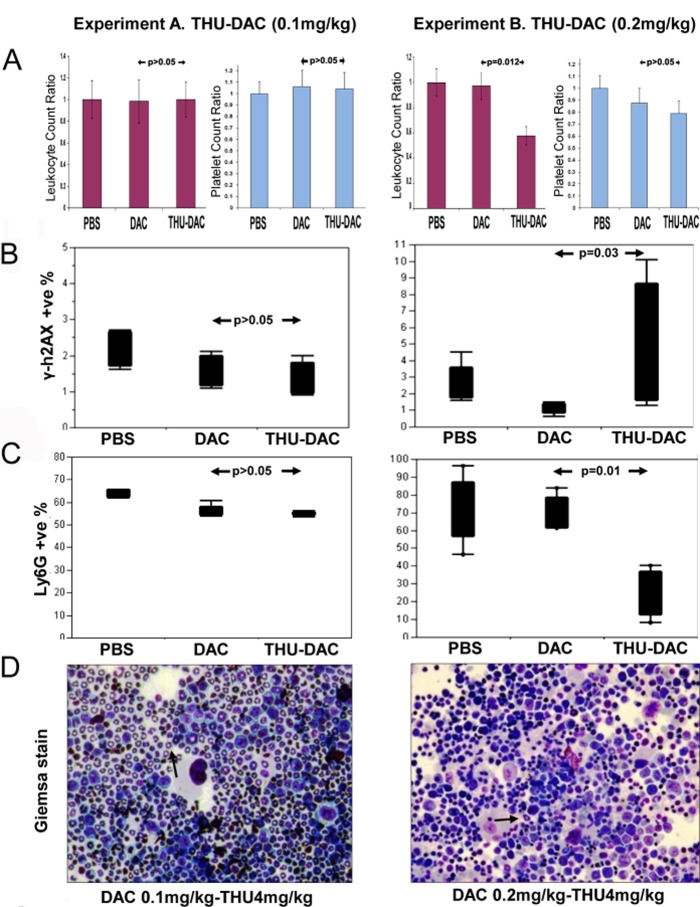
Effects of THU-DAC 0.1mg/kg versus THU-DAC 0.2mg/kg on bone marrow and peripheral blood A) There was no significant decrease in white blood cell (WBC) or platelet counts in THU-DAC 0.1 mg/kg treated mice compared to the other treatment groups (graphs show day 30/day 1 WBC and platelet ratio for individual animals). In a separate experiment (experiment B), mice (n=5/group) were treated with the same regimen of PBS or DAC, but without decreasing the dose of DAC used in combination with THU (THU-DAC 0.2 mg/kg). Peripheral blood counts were measured on treatment day 1 and 30 by tail vein phlebotomy. p-values from Wilcoxon test. B) No increase in DNA damage with THU-DAC 0.1 mg/kg as measured by flow-cytometry for phospho-H2AX in bone marrow aspirates obtained at euthanasia. C) No decrease in murine myeloid cells with THU-DAC 0.1 mg/kg as measured by flow cytometry for Ly6G (a granulocyte marker) in bone marrow aspirates obtained at euthanasia. D) Bone marrow myeloid content evaluated by Giemsa-staining of bone marrow aspirates. Giemsa-stained bone marrow aspirates. Black arrows indicate examples of murine granulocytes.

If combination therapy is to be safe and practical, ideally, it should reverse cancer cell protection from treatment without increasing toxicity to sensitive tissues such as the bone marrow. To evaluate the myelotoxicity of decitabine and THU-decitabine treatment, peripheral blood counts were measured on treatment day 1 and 30 by tail vein phlebotomy. Compared to PBS treated controls, there was no significant decrease in WBC or platelet counts in decitabine treated mice (the dose and schedule were selected for non-cytotoxic DNMT1 depletion as described previously[26]), or in mice treated with THU in combination with the decreased dose of decitabine 0.1 mg/kg (Figure 4A). However, in mice treated with THU-decitabine without decreasing the dose of decitabine (THU-decitabine 0.2 mg/kg), there was a significant decrease in white blood cell counts (Figure 4A). To further evaluate for myelotoxicity, bone marrow aspirates obtained at the time of euthanasia were evaluated by flow-cytometry for γ-H2AX, a marker of DNA damage and repair. There was no increase in γ-H2AX in bone marrow from decitabine or THU-decitabine 0.1 mg/kg treated mice, but a significant increase was noted in bone marrow from THU-decitabine 0.2 mg/kg treated mice (Figure 4B). Flow cytometric evaluation of bone marrow granulocyte content (Ly6G) and inspection of Giemsa-stained bone marrow cytospin preparations also confirmed lack of myelotoxicity with decitabine alone or THU-decitabine 0.1 mg/kg, but myelotoxicity with THU-decitabine 0.2 mg/kg (Figure 4C, D).

## DISCUSSION

At the cellular level, a number of different mechanisms have been shown to mediate resistance of cancer cells to cytidine analogues. These include downregulation of deoxycytidine kinase (DCK), the enzyme that executes the rate-limiting phosphorylation step necessary for DNA incorporation of cytidine analogues, downregulation of the nucleoside transporters that mediate cellular uptake of cytidine analogues by the pyrimidine salvage pathway, and upregulation of CDA within cancer cells (reviewed in[28]). Here, another mechanism of resistance was demonstrated: protection for otherwise treatment-sensitive cancer cells within a tissue environment that expresses high levels of CDA. This mechanism does not require adaptation or evolution by the malignancy, just preferential or incidental localization of cancer cells within a CDA-rich tissue. This mechanism could explain the very limited clinical activity of cytidine analogues against hepatotropic cancers: in clinical trials of single-agent gemcitabine to treat liver cancer, the overall response rates where 0% and 2%[9-10], even though liver cancer cells are sensitive to these drugs *in vitro*[29].

A discussion of resistance requires consideration of therapeutic index[30-31]: at relatively high concentrations (>0.5-1μM), decitabine, similar to other cytidine analogues, demonstrates anti-metabolite, DNA-damaging effects that can induce irreversible cell cycle exit by apoptosis. However, cancer cells very frequently inactivate key apoptosis-pathway genes (e.g., *TP53*, *p16/CDKN2A*) by mutation or deletion[30-31]. Hence, high anti-metabolite concentrations can have a poor therapeutic index, inducing apoptosis in normal cells (in which apoptosis genes are intact) but not necessarily proliferating cancer cells[26]. At low concentrations, however, decitabine is non-cytotoxic: unlike cytidine analogues such as cytarabine or gemcitabine, the sugar moiety in decitabine is unmodified, and decitabine can incorporate into DNA without terminating DNA chain-elongation[12-13]. These low concentrations are sufficient to deplete DNMT1 and produce a therapeutic epigenetic effect. This is because cancer cells (including cancer ‘stem’ cells[32]) express strikingly high levels of master lineage-driving transcription factors, yet have paradoxical epigenetic repression of late-maturation MYC-antagonist target genes of these transcription factors. DNMT1 depletion disrupts the chromatin-modifying enzyme network that mediates this aberrant repression, renews expression of the late-maturation MYC-antagonist genes, and triggers cell cycle exit by p53/p16-independent differentiation pathways[26, 32-35]. The same treatment maintains the self-renewal of normal stem cells, as these cells do not express high levels of lineage-specifying transcription factors needed to activate late-maturation MYC-antagonist genes[36-37]. In brief, the consequences of non-cytotoxic DNMT1 depletion are determined by the baseline maturation context, and the difference in maturation context of cancer versus normal stem cells creates a favorable therapeutic index for non-cytotoxic DNMT1 depletion by decitabine (reviewed in[32], and illustrated by efficacy without myelotoxicity in the present study). Combining THU with decitabine assists with this mode of therapy not just by interdicting sanctuary but also in other ways: THU inhibition of intestinal and hepatic CDA substantially increases oral bioavailability of decitabine. Oral administration is favored since this route of administration does not produce the high C_max_ observed with intravenous administration that can cause off-target antimetabolite effects and cytotoxicity, while the combination with THU extends the T_max_ to increase S-phase specific depletion of DNMT1[11]. Additional advantages of combining THU with decitabine are that it dampens the inter-individual variability in pharmacokinetics and pharmacodynamics caused by differences in CDA activity between individuals[11], and reverses resistance caused by upregulation of CDA expression within cancer cells[2, 7-8, 11, 34].

The molecular actions of decitabine offer an important alternative to conventional apoptosis-based chemotherapy, since low, non-cytotoxic doses can induce cell cycle exit in cancer cells by p53-independent differentiation pathways[26, 34-35, 38]. However, this relatively low dosage could be particularly vulnerable to treatment failure caused by CDA-mediated degradation, exemplified by protection for cancer cells in CDA-rich organs. The present observations demonstrate that CDA-inhibition with THU can reverse such sanctuary without necessarily increasing myelotoxicity, so long as the decitabine dosage used in combination with THU is decreased. We are actively developing oral THU-decitabine for clinical use, and a Phase 1 clinical trial is ongoing[11].
